# Neuroprotective and Anti-Inflammatory Effects of the Flavonoid-Enriched Fraction AF4 in a Mouse Model of Hypoxic-Ischemic Brain Injury

**DOI:** 10.1371/journal.pone.0051324

**Published:** 2012-12-12

**Authors:** Paul G. W. Keddy, Kate Dunlop, Jordan Warford, Michel L. Samson, Quinton R. D. Jones, H. P. Vasantha Rupasinghe, George S. Robertson

**Affiliations:** 1 Department of Pharmacology, Faculty of Medicine, Dalhousie University, Halifax, Nova Scotia, Canada; 2 Department of Environmental Sciences, Faculty of Agriculture, Dalhousie University, Truro, Nova Scotia, Canada; 3 Department of Psychiatry, Faculty of Medicine, Dalhousie University, Halifax, Nova Scotia, Canada; Rice University, United States of America

## Abstract

We report here neuroprotective and anti-inflammatory effects of a flavonoid-enriched fraction isolated from the peel of Northern Spy apples (AF4) in a mouse of model of hypoxic-ischemic (HI) brain damage. Oral administration of AF4 (50 mg/kg, once daily for 3 days) prior to 50 min of HI completely prevented motor performance deficits assessed 14 days later that were associated with marked reductions in neuronal cell loss in the dorsal hippocampus and striatum. Pre-treatment with AF4 (5, 10, 25 or 50 mg/kg, p.o.; once daily for 3 days) produced a dose-dependent reduction in HI-induced hippocampal and striatal neuron cell loss, with 25 mg/kg being the lowest dose that achieved maximal neuroprotection. Comparison of the effects of 1, 3 or 7 doses of AF4 (25 mg/kg; p.o.) prior to HI revealed that at least 3 doses of AF4 were required before HI to reduce neuronal cell loss in both the dorsal hippocampus and striatum. Quantitative RT-PCR measurements revealed that the neuroprotective effects of AF4 (25 mg/kg; p.o.; once daily for 3 days) in the dorsal hippocampus were associated with a suppression of HI-induced increases in the expression of IL-1β, TNF-α and IL-6. AF4 pre-treatment enhanced mRNA levels for pro-survival proteins such as X-linked inhibitor of apoptosis and erythropoietin following HI in the dorsal hippocampus and striatum, respectively. Primary cultures of mouse cortical neurons incubated with AF4 (1 µg/ml), but not the same concentrations of either quercetin or quercetin-3-*O*-glucose or its metabolites, were resistant to cell death induced by oxygen glucose deprivation. These findings suggest that the inhibition of HI-induced brain injury produced by AF4 likely involves a transcriptional mechanism resulting from the co-operative actions of various phenolics in this fraction which not only reduce the expression of pro-inflammatory mediators but also enhance pro-survival gene signalling.

## Introduction

Flavonoids are biologically active phenolic compounds derived from natural sources such as teas, fruits and vegetables that have attracted considerable attention for the prevention and treatment of stroke [1]. The therapeutic potential of these compounds is supported by their safety [2]–[4], efficacy in a wide variety of pre-clinical models for ischemic brain injury [5], [6] and epidemiological evidence suggesting that consumption of a flavonoid-enriched diet reduces the risk of stroke [7], [8]. More recently, associations between various flavonoid subclasses and the risk of ischemic, hemorrhagic and total stroke have been examined. Overall, these studies suggest a higher intake of flavonoids found in fruits (flavonols, flavanones and flavan-3-ols) decreases the risk of all three of these stroke outcome measures [9]–[12].

Apples are the second highest source of anti-oxidants and phenolics (next to oranges) in the North American diet [13]. The structural classes of phenolics represented in apples include flavonols (quercetin glycosides), flavan-3-ols (epicatechin, catechin), anthocyanins (cyanidin-3-*O*-galactoside), hydrochalcones (phloridzin) and phenolic acids (chlorogenic acid, cafeic acid) [14]–[17]. Apple skin contains approximately 46% of the total phenolics in apples [18], and specific flavonoids such as quercetin glycosides and cyanidin-3-*O*-galactoside are not found in the flesh of apples [17]–[22]. Extensive experimentation has demonstrated that flavonols (quercetin glycosides), flavan-3-ols (epicatechin, catechin), anthocyanins (cyanidin glycosides) and phenolic acids (chlorogenic acid) abundant in apple skin exhibit anti-oxidant, anti-inflammatory and neuroprotective properties in both *in vitro* and *in vivo* models that recapitulate the unfavourable conditions responsible for ischemic brain injury [5], [6], [23], [24]. Flavonoids belonging to these different chemical classes inhibit, to varying degrees, enzymes that phosphorylate (kinases) and dephosphorylate (phosphatases) proteins critical to signal transduction pathways which regulate oxidative stress, inflammation and cell survival [25]–[28]. The well recognized phosphatidylinositol 3-kinase inhibitor LY294002 [(4-morpholinyl)-8-phenyl-4H-1-benzopyran-4-one] is based on the chemical structure of quercetin [29], while flavopiridol [2-(2-Chlorophenyl)-5,7-dihydroxy-8-[(3R,4S)-3-hydroxy-1-methyl-4-piperidinyl]-4H-chromen-4-one] is a flavone-based inhibitor of cell cycle-dependent kinases in clinical development for the treatment of cancer [30], [31]. Flavopiridol also prevents neuronal cell loss in rodent models of cerebral ischemia [32], [33]. Subtle differences in chemical structure resulting in distinct kinase and phosphatase inhibition profiles may therefore enable flavonoid-enriched extracts to target multiple signaling pathways controlling oxidative stress, inflammation and cell survival in a complementary fashion [26], [34], [35].

In view of these findings, we have isolated and characterised the neuroprotective and anti-inflammatory properties of a flavonoid-enriched fraction from the peel of the apple cultivar Northern Spy, termed apple fraction 4 (AF4), in experimental models of ischemic brain injury. Dosage was based on the total concentration of quercetin, quercetin glycosides, catechin, epicatechin, cyanidin-3-*O*-galactoside, phloridzin, chlorogenic acid and cafeic acid that comprised AF4. The ability of oral administration of AF4 to reduce motor performance deficits and neuronal cell loss in the dorsal hippocampus and striatum was examined using a mouse model of hypoxic-ischemic (HI) brain injury. Optimal dosing parameters were established by comparing the effects of administering differing amounts of AF4 for varying periods of time prior to HI on subsequent neuronal cell loss in these structures. Quantitative RT-PCR was employed to assess the effects of AF4 on the expression of genes encoding proteins that regulate inflammation and cell survival. Lastly, we compared the effects of AF4, quercetin-3-*O*-glucoside, quercetin and major metabolites of AF4 on the survival of primary cultures of mouse embryonic cortical neurons subjected to oxygen glucose deprivation.

## Materials and Methods

### Isolation of AF4 from apple peel

The peels of the apple cultivar Northern Spy were collected from a commercial pie manufacturer, Apple Valley Foods Inc., Kentville, NS, Canada. Immediately after peeling, the peels were treated with 2% CaCl_2_ in water (w/v) at 55 ± 5°C for 10 min to prevent degradation of phenolic compounds. After draining the excess water and within 3 h of the CaCl_2_ treatment, the apple peels were transported in plastic containers to the Nova Scotia Agricultural College (NSAC). The apple peels were dried in clean plastic trays at 60±2°C for 48 h using a convection oven with air circulation (Milner Agincourt; ON, Canada). The dried peels were ground into a fine powder using a Willey mill with a 1 mm sieve screen (Model Laboratory Heavy Duty, Arthur Thomas Co.; Philadelphia, PA) and kept in a freezer (−70°C) for later use. One hundred grams of apple peel powder was placed in a 2 L flask and sonicated in 1 L of absolute ethanol twice for 15 min with a 10 min interval. The suspension was then transferred to 50 ml conical tubes and centrifuged at 3000 rpm for 15 min. The supernates of two of the above extracts (totaling 200 g of apple peel in 2 L of ethanol) were collected and evaporated to produce a 200 ml concentrate using a rotary evaporation system at 45°C (Rotavapor® R-200; Buchi, Flawil, Switzerland). The concentrated extract was made into powder using a freeze dryer (model Super Modulo, Thermo Electron Corporation, NY, US).

Flash chromatography using a sorbent (Sorbent SP207-05 Sepabeads resin brominated styrenic adsorbent; particle size 250 µm, surface area 630 m^2^/g; Sorbent Technologies; Atlanta, GA) was used to fractionate the concentrated apple peel extract described above. The apple peel extract was loaded onto a chromatography column (3.8×45 cm, Sati International Scientific Inc.; Dorval, QC, Canada) that contained 600 g of adsorbent and had been conditioned with deionized water. After loading the extract, the column was immediately washed with water (2–3 times the bed volume of water). The removal of sugar from the crude extract was monitored by measuring the Brix value of wash water using a refractometer. The washing step was terminated when the Brix value fell below 1%. The phenolic compounds retained in the column were eluted using a step gradient of ethanol (800 ml per elusion). The initial three fractions were eluted with 20%, 30% and 40% ethanol. The eight subsequent fractions were collected by increasing the volume of ethanol by increments of 5%. Eluates were concentrated to 20 ml using a rotary evaporator (Rotavapor® R-200; Buchi, Flawil, Switzerland) at 45°C. Fraction four (AF4) had the highest proportion of flavonols and other monomeric phenolic compounds, and was therefore selected for this study.

### LC-MS/MS analysis of phenolics in the C-18 fractions

Analyses of the major individual phenolic compounds present in the apple peel fractions (Table 1) were performed according to our previously described methods [36]. Analyses were performed using a Waters Alliance 2695 separations module (Waters; Milford, MA) coupled with a Micromass Quattro *micro* API MS/MS system and controlled with Masslynx V4.0 data analysis system (Micromass; Cary, NC). The column used was a Phenomenex Luna C_18_ (150 mm×2.1 mm, 5 µm) with a Waters X-Terra MS C_18_ guard column. For the separation of the flavonol, flavan-3-ol, phenolic acid and dihydrochalcone compounds, a gradient elution was carried out with 0.1% formic acid in water (solvent A) and 0.1% formic acid in acetonitrile (solvent B) at a flow rate of 0.35 ml/min. A linear gradient profile was used with the following proportions of solvent A applied at time t (min); (t, A%): (0, 94%), (9, 83.5%), (11.5, 83%), (14, 82.5%), (16, 82.5%), (18, 81.5%), (21, 80%), (29, 0%), (31, 94%), (40, 94%). The analysis of cyanidin-3-*O*-galactoside was carried out using the mobile phases of 5% formic acid in water (solvent A) and 5% formic acid in methanol (solvent B) at a flow rate of 0.35 ml/min. The linear gradient profile used was as follows; (t, A%): (0, 90%), (10, 70%), (17, 60%), (21, 48.8%), (26, 36%), (30, 10%), (31, 90%), (37, 90%).

**Table 1:** Concentration of polyphenolic compounds of fraction number 4 (AF4) determined by LC-MS/MS.

Phenolic compounds		Concentration^a^
		(µg/ml)
Flavonol	Quercetin (Q)	9.9±0.3
	Q-3-*O*-paltoside	63.8±2.4
	Q-3-*O*-rutinoside	1535.7±46.2
	Q-3-*O*-galactoside	2914.9±72.8
	Q-3-*O*-glucoside	1474.8±58.9
	Q-3-*O*-rhamnoside	2771.6±77.5
	Total Flavonols	8770.7
Anthocyanins	Cyanidin-3-*O*-galactoside	559.4±16.7
Dihydrochalcones	Phloridzin	386.8±13.6
Phenolic acids	Chlorogenic acid	1221.1±31.2
	Cafeic acid	43.6±2.0
	Total phenolic acids	1264.7
Flavan-3-ols	Catechin	106.8±3.7
	Epicatechin	1044.3±36.8
	Total Flavan-3-ol	1151.1
	Total phenolics	12132.7

a mean ± standard deviation of the mean for three determinations.

Phenolic Constituents in AF4. The total of major phenolics present in AF4 determined by LC-MS/MS to be 12132.7 µg/ml. The major polyphenolic compounds detected in AF4 belong to subclasses of flavonols, anthocyanins, dihydrochalcones, flavan-3-ols, phenolic acids and flavanols and were similar to those reported by other investigations of apple skins. The most abundant phenolics in AF4 were quercetin-3-*O*-galactoside, quercetin-3-*O*-rutinoside, quercetin-3-*O*-glucoside, quercetin-3-*O*-rhamnoside, chlorogenic acid and (−)-epicatechin.

Electrospray ionization in negative ion mode (ESI-) was used for the analysis of the flavonol, flavan-3-ol, phenolic acid and dihydrochalcone compounds. The following conditions were used: Capillary voltage of 3000 V, nebulizer gas (N_2_) temperature of 375°C and a flow rate of 0.35 ml/min. For the analysis of cyanidin-3-*O*-galactoside, electrospray ionization in positive ion mode (ESI+) was used. The settings for the positive ion experiments were as follows: Capillary voltage of 3500 V, nebulizer gas temperature of 375°C and a flow rate of 0.35 ml/min. The cone voltage (25–50 V) was optimized for each individual compound. Multiple reaction-monitoring (MRM) mode using specific precursor/product ion transitions was employed for identification and quantification of each phenolic compound using external calibration curves generated individually for each compound measured. The ion transition used for each compound were as follows: for quantification in comparison with standards: m/z 301→105 for quercetin, m/z 609→301 for quercetin-3-*O*-rutinoside, m/z 463→301 for quercetin-3-*O*-glucoside and quercetin-3-*O*-galactoside, m/z 448→301 for quercetin-3-*O*-rhamnoside, m/z 595→301 for quercetin-3-*O*-paltoside, m/z 273→167 for phloridzin, m/z 353→191 for chlorogenic acid, m/z 179→135 for cafeic acid, m/z 193→134 for ferulic acid and isoferulic acid, m/z 449→287 for cyanidin-3-*O*-galactoside, m/z 289→109 for catechin and m/z 290→109 for epicatechin. In MRM experiments, both quadrupoles were operated at unit resolution.

### Animal care

All experiments involving the use of animals were approved by the Dalhousie University Committee on Laboratory Animals (Protocol Numbers: 11-043, 11-007) and were performed in strict accordance with the guidelines for the Canadian Council on Animal Care. All surgeries were performed under 2% isoflurane at a rate of 1.5 L/min through a vaporizer. All efforts were made to minimize suffering. The animal holding rooms were on a 12-hour dark/light cycle and water and food were provided *ad libitum*.

### AF4 treatment

The AF4 dose for each experiment was standardized based on the concentration of the total non-polymeric phenolics in AF4 that included quercetin-3-*O*-glucoside, quercetin-3-*O*-galactoside, quercetin-3-*O*-rhamnoside, quercetin-3-*O*-rutinoside, epicatechin, catechin, cyanidin-3-*O*-galactoside, chlorogenic acid and phloridzin. AF4 powder was dissolved in water (5, 10, 25 or 50 mg/10 ml). The extract was administered by oral gavage to 6–8 week old C57Bl6 mice. Mice in the control group were given an equivalent volume of vehicle (water, 10 ml/kg, p.o.; once daily). Administration of water in the amount of 10 ml/kg equates to 0.25 ml for a 25 g mouse that is an acceptable volume for oral gavage. Twenty-four hours after the final dose of AF4 or vehicle, all mice were subjected to 50 min of hypoxia-ischemia (HI) and sacrificed 2 weeks later.

### HI brain injury model: Unilateral common carotid artery occlusion combined with exposure to a low oxygen environment

The procedure used to induce cerebral ischemia in adult mice was adapted from the HI method developed by Levine (1960) for rats. Mice were anaesthetized using isoflurane (Baxter Corporation; Mississauga, ON, Canada) in an induction chamber (3% vaporized with medical oxygen at a flow rate of 3 L/min). The ventral portion of the neck was shaved and then sterilized with Soluprep (SoluMed Inc.; Laval, QC, Canada) and Betadine (Purdue Frederick Inc.; Pickering, ON). Anesthesia was maintained with 2% isoflurane vaporized with oxygen at a flow rate of 1.5 L/min. A small ventral incision was made on the neck of the mouse with a pair of scissors to expose the sternohyoid and sternomastoid muscles. The left carotid artery was located beneath the intersection point of the sternohyoid and the sternomastoid muscles. The left carotid artery was carefully separated from the vagus nerve and permanently occluded using a high-temp electrocautery pen (Bovie Instruments; St. Petersburg, FL). If the common carotid artery was not completely occluded or exhibited blood loss the mouse was immediately euthanized. Following a 2–3 h recovery period the mice were placed in a hypoxia-chamber, consisting of a glass cylinder vented with 8% oxygen balanced with nitrogen flowing at a rate of 6 L/min. The chamber was placed in a water bath at 36.5°C to maintain normal body temperature. After 50 min of exposure to the low oxygen environment (8% oxygen balanced with nitrogen) mice were removed from the chamber and returned to their home cage. The mice were allowed to survive for 2 weeks following HI to permit the brain infarct in the ipsilateral hemisphere to develop before harvesting the brain tissue for histological analyses.

### Assessment of motor performance

Time spent on a rotarod (ACCURotorRotarod, ACCUScan Instruments Inc.; Columbus, OH) was measured to assess motor performance of the mice. The apparatus consists of an accelerating rotating cylinder. The rotational speed increases at a constant acceleration, thereby progressively increasing the difficulty for the mouse to maintain its balance while walking. The amount of time spent on the rod was recorded as a measure of performance, with longer times indicative of better motor performance. The acceleration of the rotarod was set to 100 rotations/min^2^. Mice were tested on the third day of vehicle (10 ml/kg, p.o.; once daily for 3 days) or AF4 (50 mg/kg, p.o.; once daily for 3 days) treatment (24 h pre-HI) and 2 weeks following HI (14 days post-HI). On each of these days the mice were tested with 3 sessions and the average time spent on the rotarod was calculated for that day. The difference in performance 14 days post-HI and 24 h pre-HI was determined and compared between the two treatment groups.

### Preparation of tissue for histology

The mice were humanely euthanized by intraperitoneal (i.p.) administration of sodium pentobarbital (Scherung-Plough; Pointe-Claire, QC, Canada) at a dose of 240 mg/kg. The mice were then transcardially perfused with 0.9% saline, followed by 4% paraformaldehyde (PFA) in phosphate buffer (pH 7.4). Brains were removed and post-fixed by storing in 4% PFA for 48–72 h. Next, the tissue was cryoprotected by submersion in a solution of 30% sucrose in 0.1 M phosphate buffer for 24 h. Free floating coronal sections were cut on a freezing microtome at a thickness of 30 µm and placed in a solution of phosphate with 0.06% sodium azide for long-term storage.

### Nissl staining

Serial forebrain sections cut 360 µm apart were mounted onto superfrost glass slides (Fisher Scientific; Nepean, ON, Canada) and allowed to dry for 24 h. Once dry, the sections were dehydrated using a graded series of increasing concentrations of ethanol (2 min of 50%, 70%, 95%, 100%) and then placed in xylenes for 5 min. Then the tissue was rehydrated using another graded series of ethanol of decreasing dilution (100%, 95%, 70%, 50%). The brain sections were then rinsed with distilled water, incubated in 1% cresyl violet solution (Sigma-Aldrich; Oakville, ON, Canada) for 10–15 min, rinsed in water again and then destained in a 1% acetic acid solution. The sections were then dehydrated through a series of graded ethanol solutions of increasing concentrations (50%, 70%, 95%, 100%) and cleared in xylenes before they were coverslipped using Cytoseal (Stephens Scientific; Riverdale, NJ).

### Infarct measurement

Volumetric measures of each hemisphere were carried out to examine the extent of hemispheric atrophy. The area of the left and right hemisphere was measured using the tracing function in Scion image on every 12^th^ section (total of 10 sections) between Bregma 1.18 mm and −2.80 mm and a volume between sections was approximated by multiplying the area by 360 µm (the distance between consecutive sections). Volumetric values from the left side of the brain were compared to the right side of the brain by generating a ‘percent of control hemisphere’ value ([left volume/right volume]/100).

### Neuronal nuclei (NeuN) immunohistochemistry

Sections were rinsed three times with phosphate buffered saline (PBS) containing 0.1% Triton X (PBS-TX) for 10 min at room temperature and then placed in 1% H_2_O_2_ in PBS-TX for 30 min to quench endogenous peroxidases. The tissue was then rinsed three times in PBS-TX for 10 min and incubated in 5% horse serum in PBS-TX for 30 min. Following incubation in serum, sections were incubated with a primary monoclonal anti-NeuN antibody raised in mouse (Cat. No. MAB377, Millipore; Etobicoke, ON, Canada) that had been diluted 1∶2000 in PBS-TX for 1 h at room temperature and then left over night at 4°C on a shaker. After incubating the tissue in primary antibody overnight the tissue was rinsed three times in PBS-TX and then incubated in a biotinylated anti-mouse secondary antibody raised in horse (Vector Laboratories Inc.; Burlingame, CA) that had been diluted 1∶500 in PBS-TX for 1 h. Following another series of washes in PBS-TX, the tissue was incubated for 1 h in an Avidin-Biotin complex diluted 1∶1000 in PBS-TX to amplify the signal from the secondary antibody. The sections were then washed and placed in a solution of 0.5 mg/ml diaminobenzidine (DAB) (Sigma-Aldrich; Oakville, ON, Canada) with nickel, glucose oxidase, ammonium chloride and D-glucose in PBS. The tissue was reacted with the DAB solution for 5–10 min until the desired staining intensity was achieved. No primary, no secondary and no ABC controls were used to confirm staining specificity. Finally, the tissue was washed and mounted onto superfrost glass slides (Fisher Scientific; Nepean, ON, Canada) and left to dry overnight. Once dry, the sections were dehydrated in a graded ethanol series of 50%, 70%, 95%, and 100%, cleared in xylenes, and coverslipped using Cytoseal (Stephen's Scientific; Riverdale, NJ).

### Image analysis of sections processed for NeuN immunohistochemistry

Sections stained for NeuN immunoreactivity from the striatum 0.1 mm anterior to bregma and the hippocampus 1.8 mm posterior to bregma were captured on a light microscope using PixeLink software at 50× (10× ocular lens and a 5× objective). The images were analyzed using ImageJ software by an observer who was blind to the treatment group of the animals. The cell counts in the striatum were determined by first converting the images to an 8-bit grey scale. The pixel threshold was set to a level three-fold greater than the background intensity and the pixels were made black on a white background by selecting the binary tool. The striatum was outlined and the analyze particle function was used to count positively labeled cells in the striatum. An index of neuronal cell survival was determined by dividing the number of NeuN positive cells in the ipsilateral striatum by the number of NeuN positive cells in the contralateral striatum: A value of 1.0 indicated no injury in the ipsilateral striatum, whereas a value less than 1.0 indicated neuronal loss. Neuronal cell loss in the hippocampus was determined similarly: The hippocampus was outlined and the area of positively labeled cells was measured with the measurement functions. Neuronal cell loss in the hippocampus was determined by measuring the area occupied by NeuN positive cells in the CA1-C3 region of brain sections cut approximately 1.8 mm posterior to bregma, as the dense packing of pyramidal neurons in the dorsal hippocampus precluded individual cell counts in sections 30 µm thick. The area of NeuN positive neurons in the ipsilateral hippocampus was divided by the area of NeuN positive neurons in the contralateral hippocampus to generate a ratio of NeuN postive cells. A value of 1.0 indicated no injury in the ipsilateral hippocampus, whereas a value less than 1.0 indicated neuronal loss.

### Total hippocampal and striatal RNA isolation and quantitative RT-PCR

Quantitative reverse-transcription polymerase chain reaction (qRT-PCR) was performed to measure the relative expression of pro-inflammatory, anti-apoptotic and erythropoietin transcripts. The dorsal hippocampus and striatum ipsilateral to the carotid artery ligation were collected from mice gavaged with vehicle (10 ml/kg, p.o.; once daily for 3 days) or AF4 (25 mg/kg, p.o.; once daily for 3 days) 1 h or 6 h following HI, submerged in RNAlater RNA stabilization reagent (Qiagen; Toronto, ON, Canada) and stored at −20°C. Total RNA was extracted from hippocampi and striati using an RNeasyLipid Tissue Mini Kit (Qiagen; Toronto, ON, Canada) according to the procedure described by the manufacturer. RNA yield and purity were measured by UV absorbance before samples were diluted to 10 ng/µL. Total RNA (50 ng) was reverse transcribed to generate first-strand cDNA and amplified using Taqman one-step EZ RT-PCR core reagents (Applied Biosystems; Foster City, CA). Mouse primers and FAM® probes were purchased pre-mixed from Applied Biosystems: Tumor Necrosis Factor-alpha (TNF-α; Mm00443258_m1), Interleukin-1 beta (IL-1β; Mm01336189_m1), Interleukin-6 (IL-6; Mm00446190_m1), Nuclear Factor of kappa Light Polypeptide Gene Enhancer in B-cells Inhibitor-alpha (IκBα; Mm00477798_m1), Toll-like Receptor 2 (TLR2; Mm00442346_m1), Toll-like Receptor 4 (TLR4; Mm00445273_m1), B-cell Lymphoma 2 (Bcl-2; Mm00477631_m1), Cellular Inhibitor of Apoptosis Protein-1 (cIAP1; Mm00431811_m1), Cellular Inhibitor of Apoptosis Protein-2 (cIAP2; Mm00431800_m1), X-linked Inhibitor of Apoptosis Protein (XIAP; Mm00776505_m1) and Erythropoietin (EPO; Mm01202755_m1). Reverse transcription, PCR amplification and fluorescence detection were performed in duplicate for each sample with an endogenous control gene (β-actin with VIC® probe: 4352341E; Applied Biosystems; Foster City, CA) using a Stratagene MX3000P instrument with MXPro software (Agilent Technologies; Santa Clara, CA). All qRT-PCR reactions were performed with a ‘no-template’ control. The relative expression of target transcripts following AF4 alone (AF4-Sham), HI (Veh-HI) or AF4 with HI (AF4-HI) was quantified using the 2^−ΔΔCT^ method [37] and expressed as fold increase in mRNA expression relative to calibrator samples extracted from mice that received vehicle and sham surgery (Veh-Sham). PCR cycling conditions were 50°C for 2 min, 60°C for 30 min, 95°C for 5 min, followed by 40 cycles of 95°C for 10 s and 60°C for 1 min. With some minor exceptions, these methods were compliant with the Minimum Information for Publication of Quantitative Real-Time PCR Experiments (MIQE) guidelines [38] as shown in Tables S1 and S2 and Figures S1 and S2.

### Preparation of mouse primary cortical neuron cultures

Embryonic day 15 timed pregnant CD1 out-bred mice were obtained from Charles River Laboratories (Charles River; QC, Canada). Primary cortical neuron cultures were prepared from cerebral cortices of wild type (WT) CD1 mouse embryos as described previously [39], with the following modifications. Pregnant CD1 females were heavily anaesthetized with isoflurane vapor (Benson Medical Industries, Inc., Markham, ON) before being euthanized by decapitation. The embryonic day 16 (E16) fetuses were immediately removed from the sacrificed pregnant females by cesarean section and placed in ice-cold Hank's Balanced Salt Solution (HBSS) (GIBCO; Invitrogen, Amarillo, CA). The meninges were removed from the brains and cortices were isolated under a dissecting microscope. The cortices from each embryo were placed in individual wells of a 24-well plate (Corning; Lowell, MA), containing 1 ml of ice-cold PBS (GIBCO; Invitrogen, Amarillo, CA) with 1 mM Mg^2+^, 13 mM glucose and 0.3% w/v bovine serum albumin (BSA) (Invitrogen, Amarillo, CA). Under sterile conditions, the tissue was briefly minced, transferred to 15 ml sterile conical tubes (Corning; Lowell, MA) and centrifuged at 350× g for 3 min at room temperature. The dissecting solution was discarded and the cortical neurons were then dissociated by incubating in 1 ml of 0.1% trypsin solution (0.1% w/v trypsin (Invitrogen, Amarillo, CA) in PBS with 1 mM Mg^2+^ and 13 mM glucose) at 37°C for 15 min. The trypsinization was inhibited by the addition of 0.5 ml of trypsin inhibitor solution that also contained DNase I (0.06% w/v trypsin inhibitor (Invitrogen; Amarillo, CA) and 0.01% DNase I (Invitrogen; Amarillo, CA) in PBS with 1 mM Mg^2+^, 13 mM glucose and 0.3% w/v BSA). The tubes were mixed briefly and the cells were centrifuged at 350× g for 3 min at room temperature. The trypsin and inhibitor solutions were discarded and each cell pellet was suspended in 1 ml of cortical neuron plating medium (Neurobasal medium (Invitrogen, Amarillo, CA) with 10% fetal bovine serum (GIBCO; Invitrogen; Amarillo, CA), 2% B27 supplement, 1 mM L-glutamine, and 1% Gentamycin (Invitrogen; Amarillo, CA), triturated 10 times and counted using trypan blue exclusion and a hemocytometer. Cortical neurons were plated in 96-well plates (Corning; Lowell, MA) that were pre-coated with poly-D-lysine (PDL; Sigma-Aldrich; Oakville, ON) according to the procedure described by the manufacturer. Briefly, plates were coated immediately before use with 100 µg/ml PDL for 5-10 min (50 µl/well), washed three times with tissue-culture grade water and left to dry for 2 h before cells were introduced. Cortical neurons were plated at a concentration of 1×10^6^ cells/ml (100 µl/well) and medium was completely changed the day after plating to serum-free cortical neuron medium (Neurobasal medium with 2% B27 supplement, 5 mM HEPES, 1 mM L-glutamine, and 1% Gentamycin), which was replaced every 3 days in culture. Cultures were maintained in a humidified, 37°C incubator with 5% CO_2_. Experiments were performed on the eighth day *in vitro* (DIV8).

### Neuroprotection assay: LDH-release

Lactate dehydrogenase (LDH) is a stable cytosolic enzyme that is released by necrotic cells upon membrane damage. The membrane integrity of cortical neurons was assayed by measuring the release of LDH using the Cytotoxicity Detection Kit^PLUS^ (Roche Applied Science; Indianapolis, IN). This assay kit detects LDH released into culture supernates by a coupled enzymatic reaction that converts a tetrazolium salt into a red formazan product. Resulting formazan was detected using an ELx800 UV spectrophotometer (Bio-tek Instruments, Inc.; Winooski, VT). Positive (100% LDH release) and negative (spontaneous LDH release) controls were prepared in triplicate according to the manufacturer's instructions. Primary cortical neuron cultures were prepared as described above. Cortical neuron cultures (DIV8) were exposed to 1 µg/ml, 0.1 µg/ml or 0.01 µg/ml of AF4, quercetin, quercetin-3-*O*-glucoside, quercetin-3′-*O*-sulphate, quercetin-3-*O*-glucuronic acid, isorhamnetin-3-glucuronic acid or the corresponding DMSO control (0.1%, 0.01% or 0.001% DMSO, respectively) in serum-free cortical neuron medium for 12 h proceeding, as well as during the 12-hour period of OGD on DIV9. Neurons were also incubated in the presence of *N* ^6^-cyclopentyladenosine (CPA; 1 µM), which has been reported to prevent cell death caused by oxygen glucose deprivation (OGD) [40]. Glucose-free medium (glucose-free Dulbecco's Modified Eagle Medium (Invitrogen; Amarillo, CA)) containing 1 µg/ml, 0.1 µg/ml or 0.01 µg/ml of AF4, quercetin, Q-3-*O*-glucoside, Q-3′-*O*-sulphate, Q-3-*O*-glucuronic acid, isorhamnetin-3-glucuronic acid or the corresponding DMSO control (0.1%, 0.01% or 0.001% DMSO, respectively) was placed in a 96-well plate and equilibrated to 0% oxygen in a modular chamber incubator (Billups-Rothenberg; Del Mar, CA). The chamber was flushed for 4 min at 20 l/min with an anoxic gas mixture (5% CO_2_ and Balanced N_2_) (PraxAIR; Dartmouth, NS) using a step-down pressure system and placed in a humidified, 37°C incubator for 12 h. Cortical neuron medium was replaced with OGD-medium (anoxic and glucose-free) and the cultures were placed in the modular chamber incubator. The chamber was flushed again with anoxic gas and placed inside a humidified, 37°C incubator for 12 h. Following, cell culture supernatants were collected on DIV9 for determination of released LDH. Absorbance was measured at 490 nm with a reference wavelength of 620 nm. Percentage of total LDH release was calculated by following the instructions provided by the manufacturer. Background was subtracted and LDH-release in each sample was expressed as a percentage of the positive control.

### Statistical analyses

Unless otherwise indicated, results are expressed as mean ± SEM (standard error of the mean). Data were analyzed using Prism 4 software for Macintosh (GraphPad Software; La Jolla, CA). Group differences were analyzed using a one-way ANOVA, and when significant, Bonferonni tests were employed for post hoc comparisons. Group differences were considered statistically significant when *p*≤0.05.

## Results

### Phenolic composition of AF4

The phenolic profile of the AF4 fraction measured by LC-MS/MS is provided in Table 1. The major groups of compounds in AF4 were flavonols, phenolic acids, flavan-3-ols, anthocyanins, and dihydrochalcones. The majority (72%) of these monomeric phenolics were quercetin glycosides.

### Oral administration of AF4 prior to HI reduces subsequent motor performance deficits and brain damage

**Figure 1:**
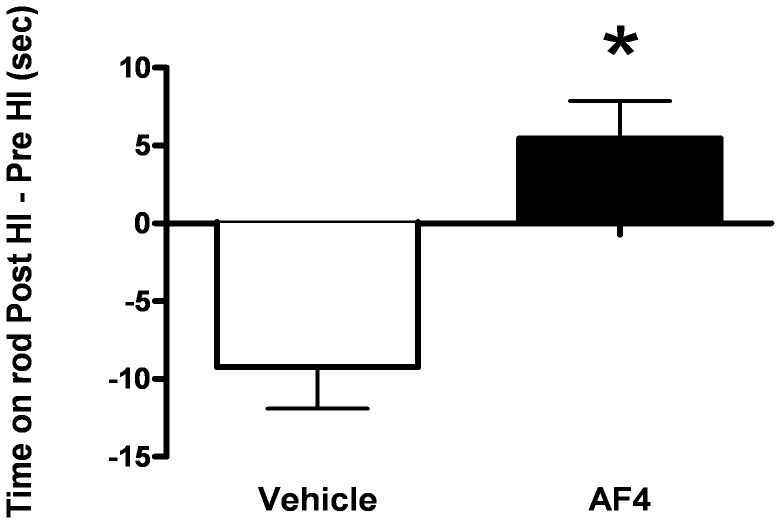
Motor performance scores. Animals were treated orally with vehicle (10 ml/kg/day for 3 days) or AF4 (50 mg/kg/day for 3 days) and subjected to 50 min of unilateral forebrain hypoxia-ischemia (HI). The amount of time spent on the rotarod was recorded as a measure of performance, with longer times indicative of better motor performance. An overall score was calculated by taking the difference between the average of 3 trials on the rotarod day 14 post-HI and 24 h pre-HI. Relative to mice that were given vehicle (10 ml/kg/day for 3 days, n = 20), animals which received AF4 (50 mg/kg/day for 3 days, n = 20) displayed superior motor performance 2 weeks after HI. *p<0.001 relative to vehicle, Mann Whitney U test (two tailed).

**Figure 2:**
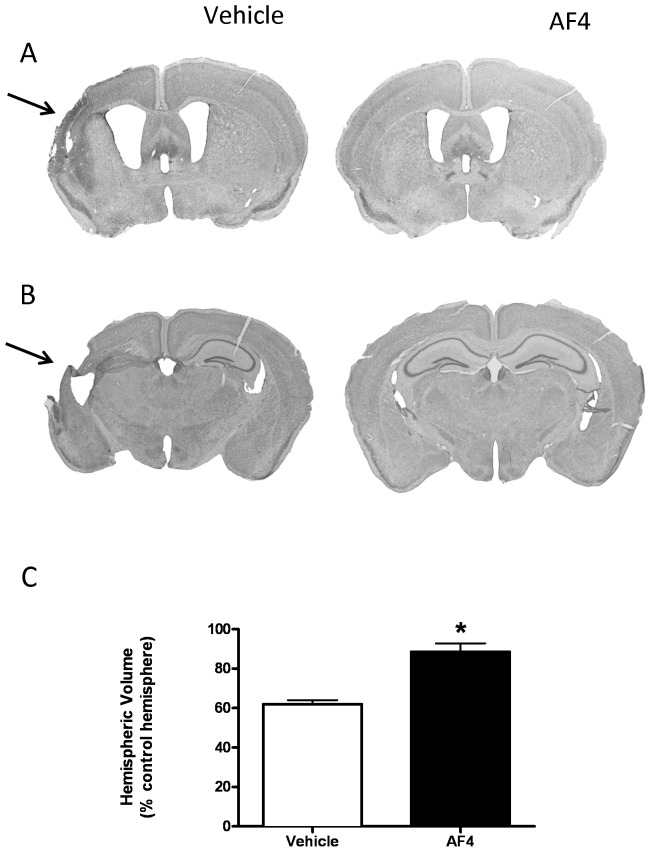
Brain injury and hemispheric volume loss. Representative Nissl-stained brain sections from two animals in the motor performance study (A and B). Note the loss of tissue (arrows) in brain sections from animals treated with vehicle (A and B, left panels) and protection produced by AF4 (A and B, right panels). Volumetric measurements from the mice used in the motor performance study (C). A Mann-Whitney U test revealed that mice which received AF4 (50 mg/kg, p.o.; once a day for 3 days) displayed considerably less brain damage than vehicle (10 ml/kg, p.o.; once a day for 3 days) treated mice. *p<0.001 relative to vehicle.

Based on the results of pilot studies, we first examined the effects of AF4 given by oral gavage at a dose of 50 mg/kg once daily for 3 days prior to HI on subsequent motor performance deficits and brain damage. Motor performance was assessed using the rotarod test in which the dependent measure was the amount of time (seconds) an animal remained on an accelerating rotating rod. Testing was performed on the third day of AF4 treatment (24 h pre-HI) and 2 weeks following HI (day 14 post-HI). The difference between rotarod scores for day 14 post-HI and 24 h pre-HI was calculated and used as a measure of motor performance (Figure 1). Mice that received vehicle (10 ml/kg, p.o.; once daily for 3 days) displayed impaired motor performance after HI (−9.23±2.68). By contrast, administration of AF4 (50 mg/kg, p.o.; once daily for 3 days) completely prevented motor performance deficits measured 14 days after HI as assessed by the rotarod test (5.47 ± 2.39). In keeping with these findings, the average hemispheric volume for animals that received AF4 (50 mg/kg, p.o.; once daily for 3 days) (88.6 ± 4.2) was greater than that for mice which were given vehicle (10 ml/kg, p.o.; once daily for 3 days) (62.0 ± 2.1) prior to HI (Figure 2).

### AF4 produces a dose-dependent reduction of HI-induced neuronal cell loss in the dorsal hippocampus and striatum

**Figure 3:**
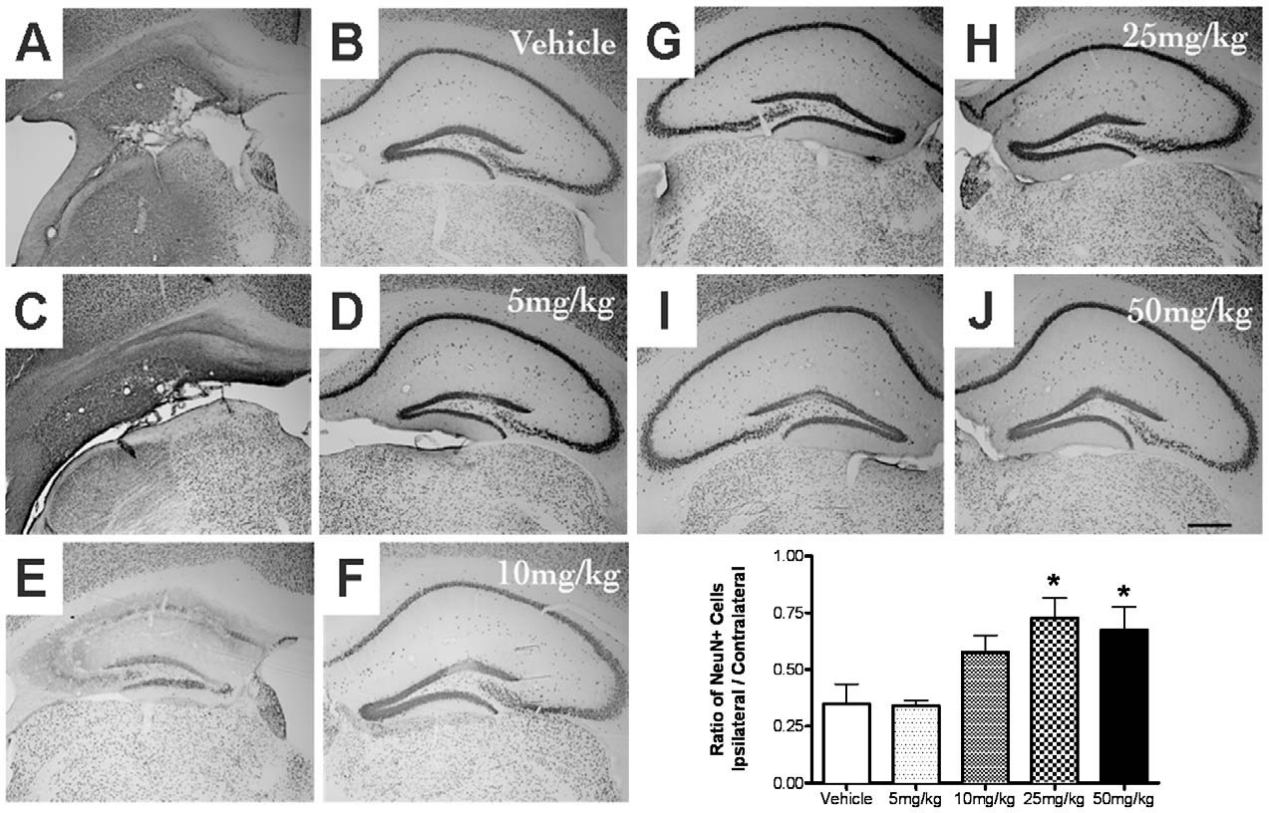
Dose-dependent reductions of HI-induced hippocampal neuron loss produced by oral administration of AF4. Five groups of adult male C57Bl/6 mice received either water (10 ml/kg, p.o.) or AF4 (5, 10, 25 or 50 mg/kg, p.o.) once daily for 3 days followed by 50 min of unilateral forebrain hypoxia-ischemia (HI) (left hemisphere, panels A, C, E, G, I) 24 h after the last dose. Animals were killed 2 weeks later and brain sections processed for immunohistochemical detection of the neuron specific marker NeuN. Cell counts revealed that neuroprotection was achieved by the 25 mg/kg dosing regime of AF4 and that increasing the dose of AF4 to 50 mg/kg did not produce a further reduction in neuronal loss in this structure (F). *p<0.05 versus vehicle and AF4 (5 mg/kg). No other comparisons were significantly different. AVONA followed by Bonferroni tests.

**Figure 4:**
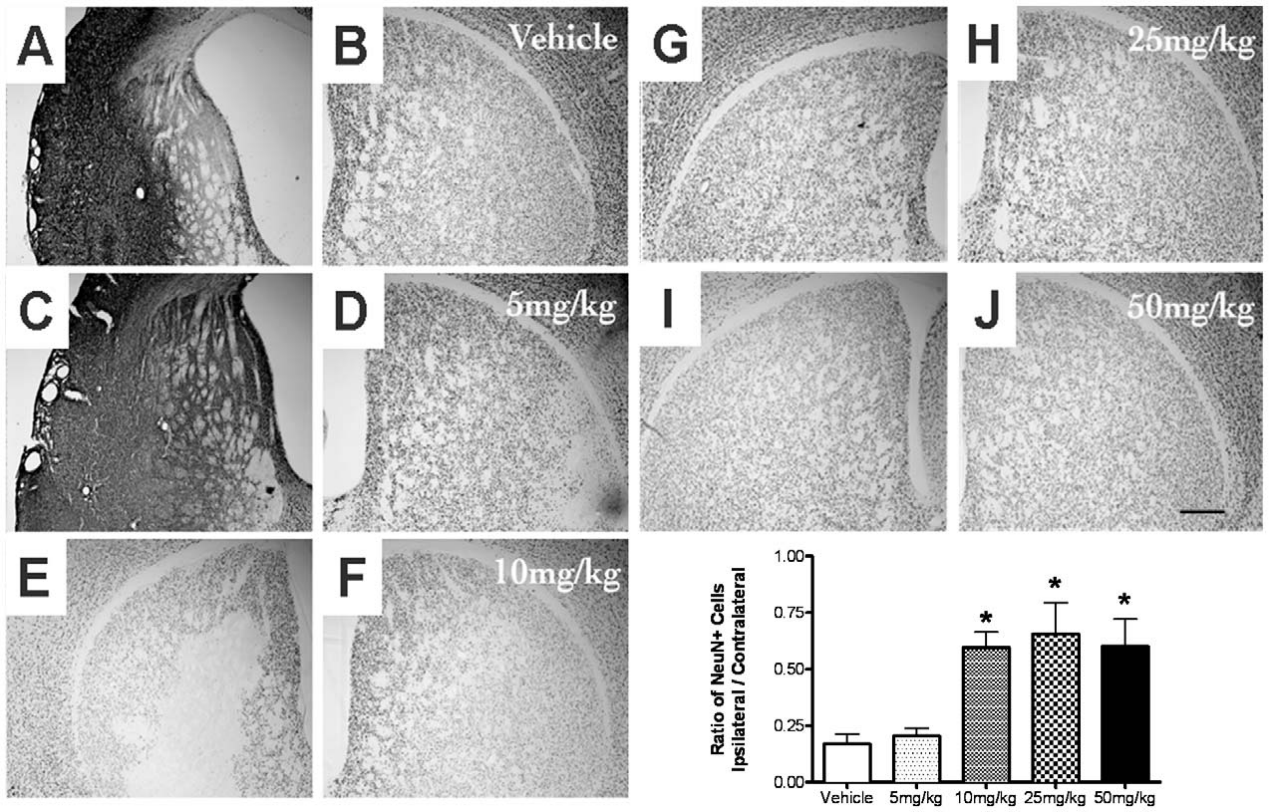
Dose-dependent reductions of HI-induced striatal neuron loss produced by oral administration of AF4. Five groups of adult male C57Bl/6 mice received either water (10 ml/kg, p.o.) or AF4 (5, 10, 25 or 50 mg/kg, p.o.) once daily for 3 days followed by 50 min of unilateral forebrain hypoxia-ischemia (HI) (left hemisphere, panels A, C, E, G, I) 24 h after the last dose. Animals were killed 2 weeks later and brain sections processed for immunohistochemical detection of the neuron specific marker NeuN. Cell counts revealed that neuroprotection was achieved by the 25 mg/kg dosing regime of AF4 and that increasing the dose of AF4 to 50 mg/kg did not produce a further reduction in neuronal loss in this structure (F). *p<0.05 versus vehicle and AF4 (5 mg/kg). No other comparisons were significantly different. AVONA followed by Bonferroni tests.

In a second experiment, we examined the dose-response relationships for the neuroprotective effects of pre-dosing with AF4 in the dorsal hippocampus and striatum of mice subjected to HI. Five groups of mice were dosed orally once daily with vehicle (10 ml/kg) or AF4 (5, 10, 25 or 50 mg/kg) for 3 consecutive days. All animals received 50 min of HI 24 h after the last administration of water or AF4. Neuronal cell loss in the hippocampus and striatum was assessed 2 weeks after HI by counting the number of NeuN positive cells in brain sections from these structures using computer-assisted image analysis. AF4 produced a dose-dependent increase in neuronal cell survival in both the dorsal hippocampus and striatum (Figures 3 and 4). For the dorsal hippocampus, the ratios of NeuN positive cells (ispilateral/contralateral sides) were 0.35±0.09 for the vehicle treatment group, 0.34±0.02 for the 5 mg/kg AF4 treatment group, 0.57±0.07 for the 10 mg/kg AF4 treatment group, 0.73±0.09 for the 25 mg/kg AF4 treatment group and 0.68±0.10 for the 50 mg/kg AF4 treatment group (Figure 3). The lowest dose of AF4 that produced an increase in neuronal cell survival in the dorsal hippocampus was 25 mg/kg (p.o., once daily for 3 days). Increasing the dose of AF4 to 50 mg/kg (p.o., once daily for 3 days) did not produce a further improvement of neuronal cell survival. The dose-response relationships for AF4-mediated neuroprotection in the striatum were similar. The ratios of NeuN positive cells were 0.17±0.04 for the vehicle group, 0.20±0.03 for the 5 mg/kg AF4 group, 0.60±0.07 for the 10 mg/kg AF4 group, 0.66±0.14 for the 25 mg/kg AF4 group and 0.61±0.12 for the 50 mg/kg AF4 group (Figure 4). The lowest dose of AF4 that produced an increase in neuronal cell survival in the striatum was 10 mg/kg (p.o., once daily for 3 days). Increasing the dose of AF4 to 25–50 mg/kg (p.o., once daily for 3 days) did not produce a further improvement of neuronal cell survival. Based on these findings, the 25 mg/kg dose of AF4 was considered optimal and was therefore selected for use in subsequent studies.

### Repeated administration of AF4 before HI is required to reduce neuronal cell loss in the dorsal hippocampus and striatum

**Figure 5:**
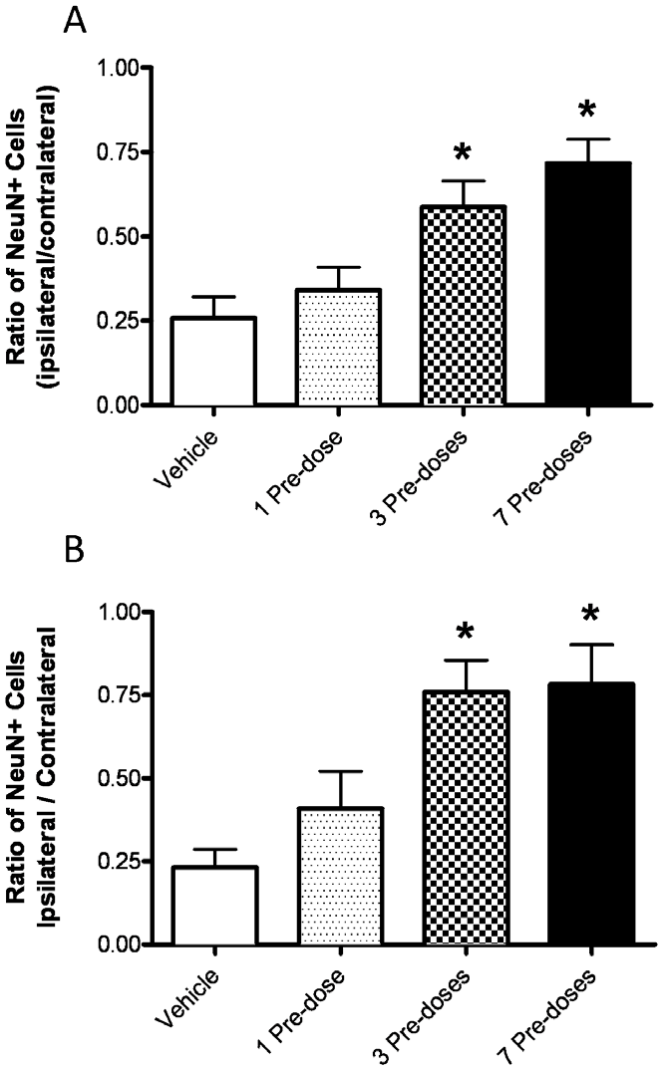
Effects of 1, 3 or 7 pre-doses of AF4 on HI-induced neuron loss in the striatum and hippocampus. Four groups, composed of 8–10 adult male C57Bl/6 mice each, were dosed orally (p.o.) once a day with water (vehicle, 10 ml/kg) or AF4 (25 mg/kg) for 1, 3, or 7 days. Twenty-four hours after the final dose of AF4 or vehicle all animals were subjected to 50 min of unilateral hypoxia-ischemia and sacrificed 2 weeks later. Brains sections from these animals were processed immunohistochemically to visualize the neuron specific marker NeuN in the striatum and hippocampus. Cell counts revealed that neuroprotection was achieved by 3 pre-doses of AF4 and that increasing the number of pre-doses to 7 did not produce a further reduction in brain injury. *p<0.05 versus vehicle and 1 pre-dose. No other comparisons were significantly different. AVONA followed by Bonferroni tests.

In a third experiment, we examined the effects of 1, 3 or 7 pre-doses of AF4 (25 mg/kg, p.o.; once per day) on the loss of NeuN positive cells in the hippocampus and striatum of animals subjected to HI (Figure 5A and B). Four groups, composed of 8–10 mice each, were dosed orally once daily with water (vehicle, 10 ml/kg) or AF4 (25 mg/kg) for 1, 3 or 7 days. All animals were exposed to 50 min of HI 24 h after the last administration of AF4 or vehicle. Administration of AF4 (25 mg/kg, p.o.) once daily for at least 3 days prior to HI was required to reduce neuronal cell loss in the dorsal hippocampus and striatum. The ratios of NeuN positive cells in the dorsal hippocampus (ipsilateral/contralateral sides) were 0.26±0.06 for the vehicle treatment group, 0.34±0.07 for the 1 pre-dose AF4 group, 0.59±0.08 for the 3 pre-dose AF4 group and 0.72±0.09 for the 7 pre-dose AF4 group (Figure 5A). Neuronal cell loss was reduced in the groups that received either 3 or 7 pre-doses of AF4 (25 mg/kg, p.o.), however, increasing the number of AF4 pre-doses from 3 to 7 did not further improve neuronal cell survival. A similar trend was observed in the striatum. The ratios of NeuN positive cells in the striatum were 0.23±0.05 for the vehicle treatment group, 0.41±0.11 for the 1 pre-dose AF4 group, 0.76±0.10 for the 3 pre-dose AF4 group and 0.78±0.12 for the 7 pre-dose AF4 group (Figure 5B). As was the case for the dorsal hippocampus, 3 and 7 pre-doses of AF4 (25 mg/kg, p.o.) produced comparable neuroprotection in the striatum. These findings suggested that repeated administration of this flavonoid-enriched fraction prior to HI was required for AF4-derived phenols to reach neuroprotective concentration and/or produce adaptive cellular responses that opposed the damaging effects of HI. To maximize the probability of observing alterations in the expression of genes that may mediate the neuroprotective effects of AF4, we therefore elected to examine the effects of administration of 7 doses of AF4 (25 mg/kg, p.o.; once daily) prior to HI in subsequent experiments.

### AF4 reduces pro-inflammatory gene expression in the dorsal hippocampus 6 h after HI

**Figure 6:**
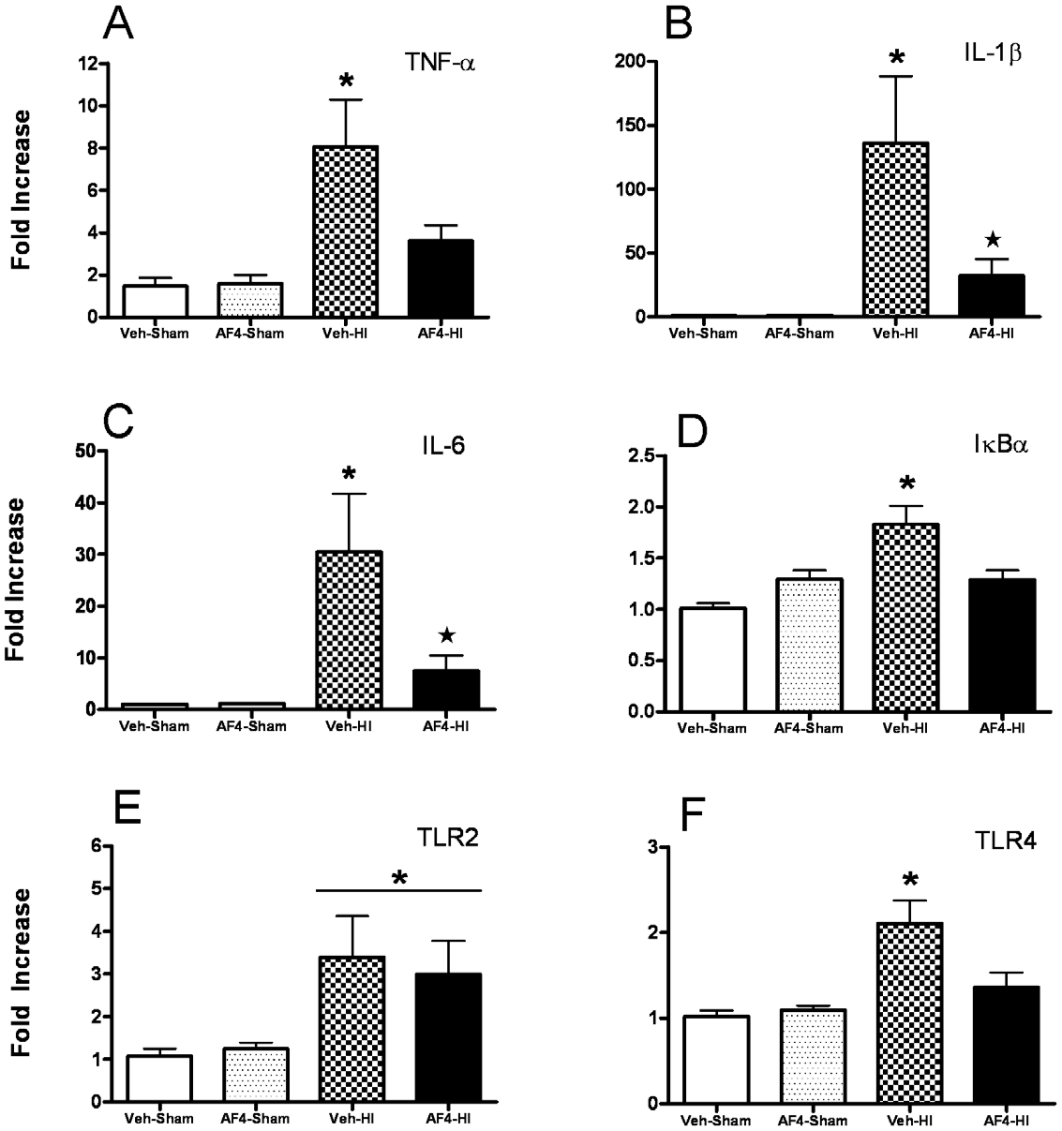
Effects of 7 days of AF4 pre-dosing on HI-induced pro-inflammatory gene expression in the hippocampus. Four groups of mice were pre-dosed with vehicle (Veh, 10 ml/kg/day for 7 days) or AF4 (AF4, 25 mg/kg/day for 7 days) and subjected to sham treatment (Sham; Veh-Sham, AF4-Sham) or unilateral forebrain hypoxia-ischemia (HI) (Veh-HI, AF4-HI) (A–F). The ipsilateral dorsal hippocampus was harvested 6 hours later. Fold increases in mRNAs encoding TNF-α, IL-1β, IL-6, IκBα, TLR2 and TLR4 were determined by qRT-PCR, n = 6 for each group. *p<0.05 relative to all other groups. ^★^p<0.05 relative to Veh-Sham and AF4-Sham. No other comparisons were significantly different. AVONA followed by Bonferroni tests.

In a fourth experiment, quantitative RT-PCR (qRT-PCR) was employed to assess the effects of AF4 (25 mg/kg, p.o.; once daily for 7 days) on the expression of genes encoding pro-inflammatory mediators. Two groups of mice, composed 12 animals each, were treated with vehicle (10 ml/kg, p.o.; once daily for 7 days) or AF4 (25 mg/kg, p.o.; once daily for 7 days). Half of the animals in these groups were exposed to 50 min of HI 24 h after the last administration of vehicle (Veh-HI) or AF4 (AF4-HI). The remaining half of these animals underwent sham surgery and served as controls (Veh-Sham and AF4-Sham). Consistent with the well established induction of pro-inflammatory cytokines by cerebral ischemia, HI produced a marked elevation of mRNAs encoding TNFα, IL-1β and IL-6 (Veh-HI; Figure 6A, B and C). The expression of these genes was reduced to levels approaching those observed in sham animals (Veh-Sham and AF4-Sham) in the AF4-HI group. Detection of IκBα mRNA levels was used as a surrogate for NF-κB activation. HI resulted in a modest but significant elevation of IκBα mRNA levels that were completely reversed in the AF4-HI group (Figure 6D).

### AF4 modulates the expression of anti-apoptotic genes following HI in a fashion that is consistent with neuroprotection

**Figure 7:**
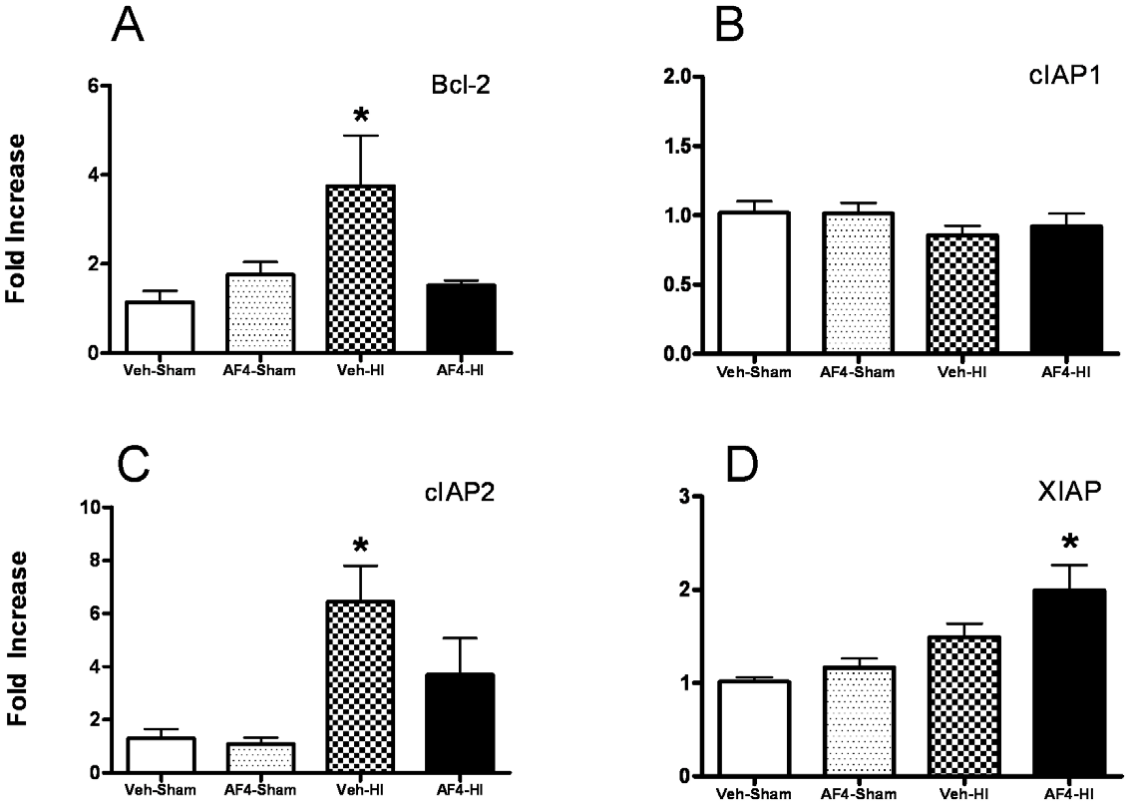
Effects of 7 days of AF4 pre-dosing on anti-apoptotic gene expression in the hippocampus following HI. Four groups of mice were pre-dosed with vehicle (Veh, 10 ml/kg/day for 7 days) or AF4 (AF4, 25 mg/kg/day for 7 days) and subjected to sham treatment (Sham; Veh-Sham, AF4-Sham) or unilateral forebrain hypoxia-ischemia (HI) (Veh-HI, AF4-HI) (A–D). The ipsilateral dorsal hippocampus was harvested 6 hours later. Fold increases in mRNAs encoding Bcl-2, cIAP1, cIAP2 and XIAP were determined by qRT-PCR, n = 6 for each group. A,*p<0.05 relative to all other groups. C and D, *p<0.05 relative to Veh-Sham and AF4-Sham. No other comparisons were significantly different. AVONA followed by Bonferroni tests.

Using RNA from the previous experiment, we next examined the effects of AF4 on expression of genes encoding the anti-apoptotic proteins Bcl-2, cIAP1, cIAP2 and XIAP in the dorsal hippocampus 6 hr following HI (Figure 7A–D). HI increased mRNA levels for Bcl-2 and cIAP2 (Veh-HI) that were reduced to values observed in the sham surgery groups (Veh-Sham and AF4-Sham) by administration of AF4 (25 mg/kg, p.o.; once daily for 7 days) before HI (AF4-HI) (Figure 7A and C). Unlike the gene expression profiles for these anti-apoptotic genes, XIAP mRNA levels were elevated in the AF4-HI group relative to Veh-HI animals. No differences in cIAP1 expression were detected between the four groups (Figure 7B).

### Effects of AF4 on erythropoietin gene expression in the dorsal hippocampus and striatum after HI

**Figure 8:**
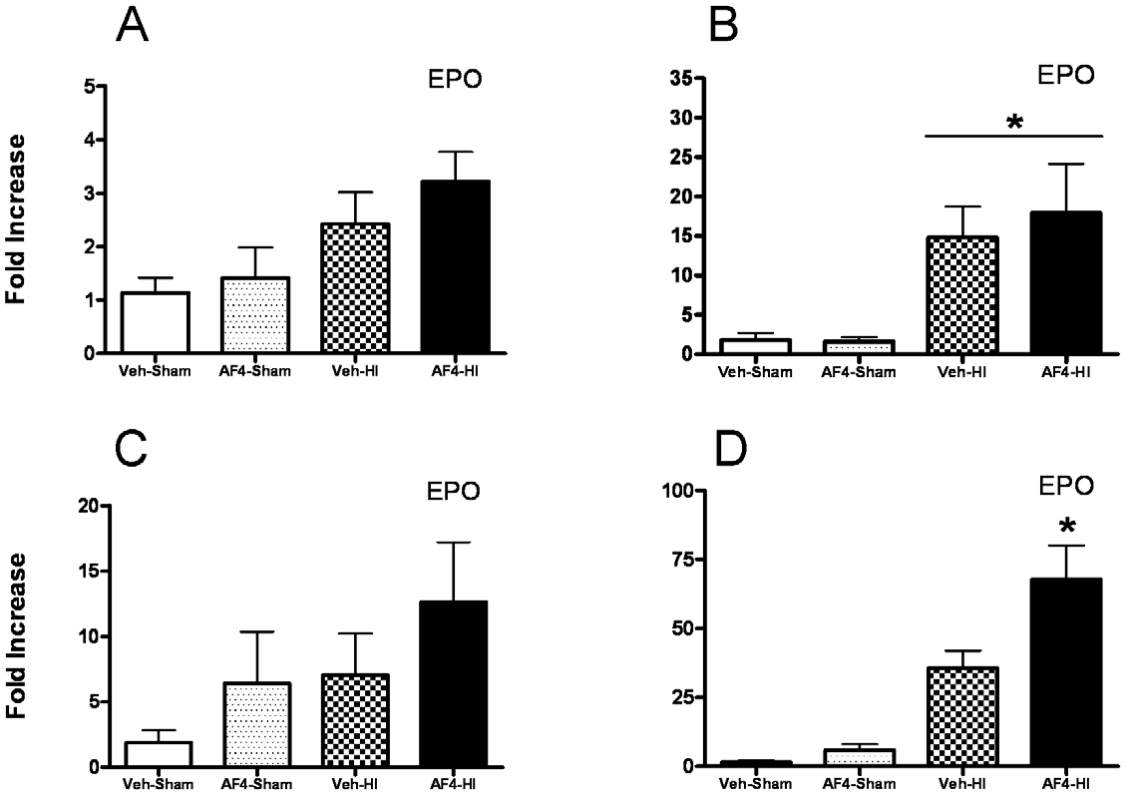
Effects of 7 days of AF4 pre-dosing on erythropoietin gene expression in the hippocampus and striatum 1 or 6 h following HI. Four groups of mice were pre-dosed with vehicle (Veh, 10 ml/kg/day for 7 days) or AF4 (AF4, 25 mg/kg/day for 7 days) and subjected to sham treatment (Sham; Veh-Sham, AF4-Sham) or unilateral forebrain hypoxia-ischemia (HI) (Veh-HI, AF4-HI) (A–D). The ipsilateral dorsal hippocampus (A and B) and striatum (C and D) were harvested from these animals 1 h (A and C) or 6 h (B and D) later. Fold increases in mRNAs encoding erythropoietin (EPO) were determined by qRT-PCR, n = 6 for each group. B and D, *p<0.05 relative to all other groups. ^★^p<0.05 relative to all other groups. No other comparisons were significantly different. AVONA followed by Bonferroni tests.

In a fifth experiment, we compared the effects of AF4 (25 mg/kg, p.o.; once daily for 7 days) on expression of the gene encoding erythropoietin (EPO) in the dorsal hippocampus and striatum 1 and 6 h after HI. Two groups of mice, composed of 24 animals each, were treated with vehicle (10 ml/kg, p.o.; once daily for 7 days) or AF4 (25 mg/kg, p.o.; once daily for 7 days). Half of the animals in these groups were exposed to 50 min of HI 24 h after the last administration of vehicle (Veh-HI) or AF4 (AF4-HI). The remaining half of these animals underwent sham surgery and served as controls (Veh-Sham and AF4-Sham). Total RNA was extracted from the dorsal hippocampus and striatum 1 and 6 h after HI. There was a clear statistical trend for the induction of EPO gene expression by HI in the dorsal hippocampus at 1 h (Figure 8A, ANOVA, p = 0.065) that reached significance at 6 h (Figure 8B). Although no statistical differences were detected 1 h after HI in the striatum, AF4 administration appeared to enhance EPO mRNA levels in animals that received HI (AF4-HI) (Figure 8C). At 6 h, EPO mRNA levels were clearly elevated in animals subjected to HI (Veh-HI and AF4-HI) relative to sham surgery controls (Veh-Sham and AF4-Sham). Moreover, AF4 administration further enhanced levels of EPO mRNA in animals subjected to HI (AF4-HI) (Figure 8D).

### AF4 reduces the death of primary cultures of mouse cortical neurons subjected to oxygen glucose deprivation (OGD)

**Figure 9:**
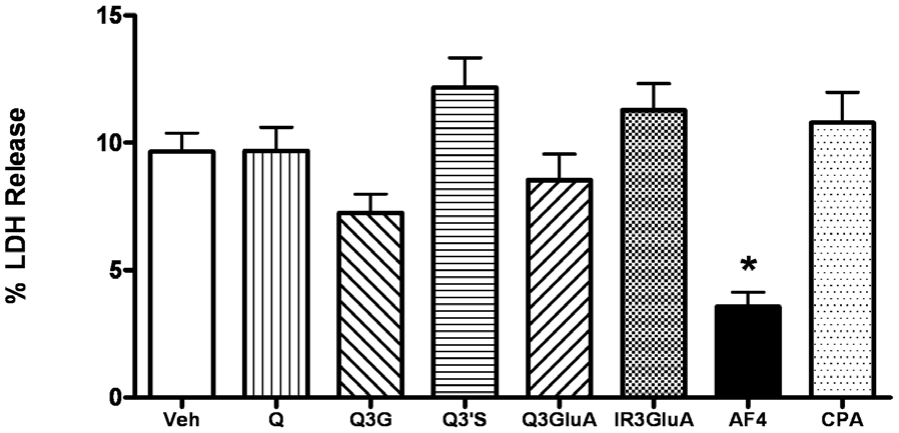
AF4 attenuated OGD-induced death of mouse primary cortical neuron cultures. Percentage of total possible LDH release from mouse primary cortical cultures treated with vehicle (0.1% DMSO), quercetin, quercetin-3-*O*-glucoside (Q3G) or quercetin metabolites [Q-3′-*O*-sulphate (Q3′S), Q-3-*O*-glucuronic acid (Q3GluA), isorhamnetin-3-glucuronic acid (IR3GluA)] or AF4 (n = 12) at a concentration of 1.0 µg/ml and subjected to 12 h of OGD. Treatment with AF4 but not quercetin, quercetin metabolites or cyclopentyl adenosine (CPA) showed a significant reduction in % LDH release in comparison to the vehicle treatment group at a concentration of 1.0 µg/ml. *p<0.05 versus all other groups. No other comparisons were significantly different. AVONA followed by Bonferroni tests.

In a sixth and final experiment, the neuroprotective effects of AF4, quercetin, quercetin-3-*O*-glucoside and several major quercetin metabolites (quercetin-3′-*O*-sulphate, quercetin-3-*O*-glucuronic acid, isorhamnetin-3-glucuronic acid) relative to vehicle were examined using primary cultures of mouse cortical neurons subjected to OGD. Mouse primary cortical neuron cultures (DIV8 cells, E16 Cortical Cultures) were incubated with either 1 µg/ml, 0.1 µg/ml or 0.01 µg/ml of AF4, quercetin, quercetin-3-*O*-glucoside (Q3G), quercetin-3′-*O*-sulphate (Q3′S), quercetin-3-*O*-glucuronic acid (Q3GluA), isorhamnetin-3-glucuronic acid (IR3GluA) or the corresponding DMSO control (0.1%, 0.01% or 0.001% DMSO, respectively) for 12 h proceeding, as well as during the 12-hour period of OGD on DIV9. Neurons were also incubated in the presence of CPA (1 µM) for equivalent periods of time. The percent of total possible LDH (100% cell death) release into serum free/aglycaemic/anoxic (OGD) medium was determined and used as a measure of total cell death. Treatment with AF4, quercetin, quercetin-3-*O*-glucoside or quercetin metabolites at concentrations of 0.01 or 0.1 µg/ml did not reduce % LDH release in comparison to the vehicle treatment group (data not shown). By contrast, treatment with AF4, but not quercetin quercetin-3-*O*-glucoside or quercetin metabolites, at a concentration of 1.0 µg/ml produced a 65% reduction in LDH release in comparison to the vehicle treatment group (Figure 9). These data demonstrate that AF4, but not quercetin, quercetin-3-*O*-glucoside or quercetin metabolites, directly protected mouse primary cortical neurons from OGD-induced neuronal cell loss.

## Discussion

### AF4 reduces neuronal cell loss and motor performance deficits in a mouse model of HI brain injury: Dose-response relationships and pre-treatment effects

The primary finding of this study was the ability of oral pre-dosing with AF4 (25 or 50 mg/kg, once daily for 3 days) to reduce neuronal cell loss in the dorsal hippocampus and striatum of mice subjected to a model of HI-induced brain damage. The striatum is a structure in the basal ganglia that plays an important role in the control of movement [41]. The preservation of motor performance in animals that received AF4 (50 mg/kg, once daily for 3 days) before HI therefore indicates that surviving neurons in this structure operated properly. Dose-response studies revealed that the lowest dose of AF4 that produced maximal neuroprotection in the dorsal hippocampus and striatum was 25 mg/kg (once daily for 3 days). This dose was used to determine the effects of administering AF4 (25 mg/kg, p.o.; once daily) 1, 3 or 7 times prior to HI on neuronal cell survival in these structures. At least 3 days of dosing with AF4 (25 mg/kg, p.o.; once daily) before HI was required to reduce neuronal cell loss in the dorsal hippocampus and striatum. At least two mechanisms may account for the necessity to pre-treat animals with AF4 (25 or 50 mg/kg, p.o.) for at least 3 days to prevent motor performance deficits and neuronal cell loss in the HI model. First, 3 days of pre-dosing may be required for AF4-derived phenols and/or their active metabolites to reach physiological concentrations that are neuroprotective. Second, several days of pre-dosing with AF4 may be necessary to produce adaptive changes in gene expression that enable various cell types within the brain to resist the injurious effects of HI. This second explanation is supported by DNA microarray and protein profiling studies demonstrating that flavonoid-mediated neuroprotection is closely associated with adaptive changes in the expression of many genes predicted to increase resistance to ischemic brain damage [42]–[49].

### AF4 modulates pro-inflammatory gene expression in a fashion consistent with neuroprotection in the dorsal hippocampus

HI produced a robust increase in the expression of genes encoding the pro-inflammatory mediators IL-1β, TNF-α and IL-6 in the dorsal hippocampus that was suppressed by pre-treatment with AF4 (25 mg/kg, p.o.; once daily for 3 days). Given the injurious role played by pro-inflammatory cytokines in ischemic brain damage [50], [51], these findings suggest that AF4 may reduce neuronal cell loss in part by decreasing the production of IL-1β, TNF-α and IL-6. Induction of the transcriptional regulating factor NF-κB drives the expression of many of the pro-inflammatory mediators that contribute to ischemic brain damage [50]–[53]. Termination of NF-κB transcription is mediated by NF-κB-dependent synthesis of the inhibitory IκBα subunit [54], [55]. Measurement of IκBα mRNA levels by qRT-PCR has been shown to be a sensitive and reliable method to quantify the transcriptional power of NF-κB [56]. Consistent with a role for NF-κB in the transcriptional activation of genes encoding IL-1β, TNF-α and IL-6, IκBα mRNA levels were also increased in the dorsal hippocampus of animals that received vehicle (10 ml/kg; once daily for 3 days) prior to HI. Similarly, the suppression of HI-induced cytokine gene expression by AF4 (25 mg/kg, p.o.; once daily for 3 days) was mirrored by a reduction in IκBα mRNA levels. *In vitro* studies support this observation by demonstrating that AF4-derived flavonoids such as epicatechin and quercetin block distinct signal transduction events necessary for NF-κB activation [57]–[60]. Further neuroprotective benefits of AF4 pre-treatment in the context of ischemic brain injury may be derived from the direct inhibition of NF-κB induction within neurons that would otherwise drive the expression of pro-apoptotic genes such as p53 [61]–[64]. Lastly, we examined the effects of AF4 (25 mg/kg, p.o.; once daily for 3 days) on the expression of toll-like receptors TLR2 and TLR4 following HI. Cerebral ischemia increases the central expression of both TLR2 and TLR4 that play opposite roles in the modulation of ischemic brain injury. Studies that have utilized mice which lack either TLR2 or TLR4 suggest that, following an experimental stroke, increased TLR2 signaling enhances resistance to ischemic brain damage, whereas TLR4 activation aggravates the injurious effects of cerebral ischemia [65]–[67]. These findings are supported by the neuroprotective effects of the TLR2 agonist Pam3CSK4 in a mouse model of transient neocortical focal ischemia [68]. In agreement with these distinct roles for TLR2 and TLR4 in the modulation of ischemic brain damage, we found that pre-treatment with AF4 (25 mg/kg, p.o.; once daily for 3 days) preferentially decreased the induction of TLR4 mRNA levels by HI. The preservation of HI-induced increases in TLR2 mRNA levels in the dorsal hippocampus of animals that received AF4 (25 mg/kg, p.o.; once daily for 3 days) is therefore consistent with a neuroprotective role for TLR2. Similar findings have recently been reported for the flavonoids baicalin and luteolin that reduced infarct volume and neurological deficits in a manner associated with a down-regulation of mRNA and protein levels for TLR4 and NF-κB in a model of permanent focal ischemia [69], [70].

### AF4 alters the expression of anti-apoptotic genes following HI

The neuroprotective effects of flavonoids have been linked to activation of pro-survival signaling mediated by the PI3/Akt and Erk pathways that stimulate expression of the proto-typical anti-apoptotic genes Bcl-2 and XIAP [71]–[78]. HI enhanced the expression of mRNA encoding Bcl-2 in the dorsal hippocampus that was completely reversed by AF4 (25 mg/kg, p.o.; once daily for 3 days). In situ hybridization histochemical studies have localized increases in Bcl-2 mRNA following transient global cerebral ischemia to pyramidal neurons in the hippocampus that are exquisitely sensitive to ischemic injury [79]. Energy depletion resulting from cerebral ischemia causes the excessive release of glutamate and over-activation of post-synaptic NMDA receptors [80]–[82]. This in turn triggers a massive rise in intracellular calcium that activates the transcriptional regulating factor CREB [83], [84]. CREB is known to mediate transcriptional activation of Bcl-2 in many cell types including neurons after cerebral ischemia [85]. Reversal of HI-induced increases in Bcl-2 gene expression by AF4 may therefore reflect a reduction in NMDA receptor-mediated CREB signaling. This hypothesis is supported by the ability of intravenous administration of a polyphenolic enriched-extract from red wine to reduce both brain damage and the burst of glutamate release, as assessed by *in vivo* brain microdialysis, produced by transient focal ischemia [86]. HI also increased mRNA levels for cIAP2 in the dorsal hippocampus. As was the case for HI-induced Bcl-2 gene expression, these increases were also attenuated by pretreatment with AF4 (25 mg/kg, p.o.; once daily for 3 days). Hypoxia is a well accepted trigger for cIAP2 expression that following induction protects epithelial cells from the injurious effects low oxygen levels [87]. Cytokines induced by cerebral ischemia such as TNF-α and granulocyte colony-stimulating factor also increase cIAP2 protein levels and thus enhance the apoptotic resistance of vascular endothelial cells and neurons [88]–[92]. In primary cortical neurons, exposure to high concentrations of ATP (100 µm, 30 min) reached following cerebral ischemia enhances cIAP2 expression via the JAK-Stat3 pathway [93]. Taken together, these findings suggest that decreased cIAP2 expression in animals that received AF4 may reflect the ability of this flavonoid-enriched fraction to reduce both the expression of pro-inflammatory cytokines and extracellular concentrations of ATP after HI. Unlike Bcl-2 and cIAP2 gene expression that was induced by HI, mRNA levels for XIAP were unchanged 6 h after HI. However, relative to animals that received vehicle (10 ml/kg, p.o.; once daily for 3 days) before HI, mice treated with AF4 (25 mg/kg, p.o.; once daily for 3 days) prior to this insult displayed a small but significant rise of mRNA levels for this potent anti-apoptotic protein. The neuroprotective effects of quercetin against status epilepticus-induced hippocampal neuronal cell loss in rats are also associated with elevated XIAP mRNA and protein levels in this brain region [94]. Furthermore, sex differences in the susceptibility of mice to ischemic brain damage are conferred by microRNA-mediated translational arrest of XIAP such that females, which are more resistant to this form of injury than males, have higher brain levels of XIAP mRNA [95]. These findings suggest that the ability of AF4 to increase XIAP gene expression after HI may be functionally relevant to the neuroprotective effects of this flavonoid-enriched fraction.

### AF4 enhances the induction of EPO gene expression by HI

EPO gene expression is activated by hypoxia as part of the adaptive cellular response to reduced oxygen supply [96], [97]. Molecular oxygen is utilized by prolyl hydroxylases to catalyze the hydroxylation of the alpha subunits for the transcriptional regulating factors Hypoxia Inducible Factor (HIF)-1 and HIF-2 [98]–[100]. Once hydroxylated, HIF-1α and HIF-2α are targeted for degradation by the proteosome [101], [102]. Reduced oxygen levels result in stabilization of HIF-1α and HIF-2α and the activation of hypoxia-responsive genes. Although HIF-1 was identified first it is now well recognized that hypoxia-induced increases in EPO gene expression are mediated by HIF-2α [103], [104]. Quercetin, a major flavonoid component of AF4, has been shown to inhibit prolyl hydroxylase activity resulting in the stabilization of HIF-1α and HIF-2α and the activation of hypoxia response genes [105]–[108]. In keeping with these findings, AF4 pretreatment further enhanced HI-induced increases in EPO mRNA expression in the striatum. Quercetin has also been shown to enhance angiogenesis [106], while consumption of epicatechin elevates hippocampal angiogenesis and the retention of spatial memory in mice [109]. The neuroprotective effects of red wine-derived phenolics in a rat model of transient focal ischemia are also associated with increased brain artery diameter [86]. These studies raise the possibility that increased cerebral vascularization resulting in improved oxygen delivery may contribute to the neuroprotective effects of AF4. If this protective mechanism had been in operation, we would have expected expression of EPO, a hypoxia responsive gene, to be reduced in the dorsal hippocampus and striatum of animals that received AF4 before HI. However, this was not the case. HI-induced increases in EPO expression were unaltered in the dorsal hippocampus and enhanced in the striatum of animals that received AF4. This strongly suggests that AF4 did not improve neurological outcome after HI by enhancing oxygen delivery to the brain. EPO has well established neuroprotective effects in models of ischemic and traumatic brain injury suggesting that this cytokine may have reduced neuronal loss in the striatum of animals treated with AF4 [110]–[113]. In view of the ability of EPO to increase XIAP levels in neurons [114], [115], it also tempting to speculate that increased levels of this cytokine may have mediated the induction of XIAP gene expression by AF4 in animals which received HI.

### Neuroprotective and anti-inflammatory effects of the phenolic constituents of AF4

The major flavonoid constituents of AF4 are the quercetin glycosides (quercetin-3-*O*-galactoside, quercetin-3-*O*-rutinoside, quercetin-3-*O*-glucoside and quercetin-3-*O*-rhamnoside) that account for about 70% of the total phenolic content of this fraction (Table 1). Numerous studies have reported that the administration of these constituents individually (quercetin aglycone, quercetin-3-*O*-rutinoside or quercetin-3-*O*-galactoside) or as a mixture derived from natural sources (*Fagopyrum esculentum*, *Abelmoschus manihot*) reduce brain damage and neurological deficits in rodents following cerebral ischemia [93], [116]–[122]. Because quercetin, quercetin-3-*O*-rutinoside and quercetin-3-*O*-galactoside have low oral bioavailability [15], [123], [124], the neuroprotective effects of these compounds are typically examined following injection by the intraperitoneal or intravenous route (for review see [125]). Nevertheless, prolonged oral administration of quercetin-3-*O*-rutinoside (25 mg/kg; once daily for 21 days) prior to an experimental stroke produced by transient occlusion of the middle cerebral artery has been found to reduce infarct size and neurological deficits [116]. To the best of our knowledge, the neuroprotective effects of quercetin-3-*O*-rhamnoside and quercetin-3-*O*-glucoside have not been examined individually using an animal model of ischemic brain damage. However, quercetin-3-*O*-rhamnoside as well as quercetin and quercetin-3-*O*-rutinoside exhibit neuroprotective properties in an *ex vivo* model of methylmercury-induced neurodegeneration [126]. These observations coupled with the high concentration of several quercetin glycosides in AF4 suggest that all of them may contribute to the neuroprotective effects of this flavonoid-enriched fraction. The next most abundant phenolic in AF4 is chlorogenic acid (about 10% of the total phenolic content, Table 1). Chlorogenic acid has well established anti-oxidant, anti-inflammatory and neuroprotective properties [24], [127], which are thought to account for its ability to reduce behavioural deficits in a rabbit model of embolic stroke [128]. The flavan-3-ols (catechins), represented here by epicatechin and catechin, composed just less than 10% of the total phenolic content of AF4. Nevertheless, oral administration of epicatechin or catechin protects against ischemic brain damage suggesting these compounds may contribute to the neuroprotective effects of AF4 [129], [130]. Finally, the anthocyanin cyanidin-3-*O*-galactoside and dihydrochalcone phloridzin were responsible for the remaining 7% of the total phenolic content for AF4. To the best of our knowledge, the neuroprotective effects of cyanidin-3-*O*-galactoside or phloridzin in a model of cerebral ischemia have not been reported, however, oral administration of cyanidin-3-*O*-glucoside shortly after transient focal ischemia produces a modest reduction in infarct volume [131], [132]. The post-dosing efficacy of cyanidin-3-*O*-glucoside [132] and quercetin [119] in focal models of stroke suggest that administration of AF4 shortly after cerebral ischemia might also improve functional outcomes such as motor deficits and infarct size.

### Flavonoid constituents of AF4: Bioavailability and pharmacokinetic properties in rodents and humans

In relating our findings to humans, it is important to consider the bioavailability and pharmacokinetic properties of flavonoids that comprise AF4 in animals and humans. Following ingestion, flavonoids undergo extensive transformation resulting in metabolites with distinct pharmacodynamic and pharmacokinetic properties [35], [133]. In both rodents and humans, quercetin glycosides (quercetin-3-*O*-glucoside) and catechins (catechin and epicatechin) are better absorbed than galloylated catechins (epigallocatechin) and anthocyanins (cyanidin) [124], [133]–[135]. The long *in vivo* half-life of quercetin and catechin metabolites in rodents, dogs and humans favours plasma accumulation with repeated dosing [124], [133], [136]. This may account in part for the necessity to dose mice with AF4 for at least 3 days prior to HI in order to achieve neuroprotection. Furthermore, several *in vivo* metabolites of quercetin and catechins exert anti-oxidant, anti-inflammatory and neuroprotective activities [35]. Taken together, these findings not only support the importance of quercetin glycosides and catechins for the neuroprotective and anti-inflammatory effects of AF4 in mice but also their relevance for humans.

### Possible synergistic interactions between flavonoids that comprise AF4

The neuroprotective effects of AF4 were also examined by determining whether primary cultures of mouse cortical neurons treated with this fraction were rendered more resistant to death produced by oxygen-glucose deprivation (OGD) than cultures that received vehicle. This model is known to induce the death of neurons by a variety of pathogenic mechanisms implicated in neurodegenerative disorders such as stroke, Alzheimer's disease, multiple sclerosis and Parkinson's disease including excitotoxicity, oxidative stress, calcium over-load, protease activation and apoptosis [137], [138]. Relative to primary cultures of mouse cortical neurons treated with vehicle, addition of AF4 (1 µg/ml) to mouse cortical cultures reduced OGD-induced cell death by over 60%. This finding raises the possibility that the neuroprotective effects of AF4 in the HI model may be mediated by direct effects of this fraction on the brain. In support of this hypothesis neither quercetin nor quercetin-3-*O*-glucoside, nor several major AF4 metabolites (quercetin-3-*O*-sulphate, quercetin-3-*O*-glucuronic acid or isorhamnetin-3-glucuronic acid) protected cortical neurons against OGD at a concentration of 1 µg/ml. These results also suggest that the ability of AF4 to protect cortical neurons against OGD may derive from the combined effects of multiple flavonoids contained within this fraction that work in synergy. For instance, compared to the present experiment, a much higher concentration of quercetin (40 µg/ml) has been reported to be only modestly protective (30% decrease in cell death) against a considerably shorter period of OGD (50 min) [139]. In the case of cyanidin-3-*O*-β-D-glucopyranoside (10 µg/ml), concentrations 10 times higher than that of AF4 (1 µg/ml) reduced cell loss by only 15%, resulting from a short period of OGD (2 h versus 12 h used by the present study) [131]. Neuroprotection produced by the flavones baicalin and luteolin in the OGD model also require concentrations that exceed 10 µg/ml [140]–[142]. In other models of oxidative stress-induced cell death such as that produced by exposure to hydrogen peroxide [143] or oxidized low-density lipoproteins [144], protection only occurs at 10–15 fold greater concentrations (10–15 µg/ml) for individual flavonoids than AF4 (1 µg/ml). Nevertheless, by combining different flavonoids such as catechin (5 µg/ml), epicatechin (5 µg/ml) and epigallocatechin gallate (0.5 µg/ml) it is possible to see benefits on biochemical correlates of cell injury in the OGD model that are not observed with 2–10 fold greater concentrations of these flavonoids individually [145]. Taken together, these findings suggest that the high neuroprotective potency of AF4 may be derived from optimization of the flavonoid composition necessary to prevent the deleterious effects of OGD.

## Conclusions

In summary, we have demonstrated that repeated oral administration of the flavonoid-enriched fraction AF4 (25–50 mg/kg, once daily for 3 days) prior to an experimental stroke produced by unilateral forebrain hypoxia-ischemia prevents motor performance deficits and markedly attenuates neuronal cell loss in the dorsal hippocampus and striatum. These profound neuroprotective effects were associated with a near complete suppression of HI-induced increases in mRNA levels for genes encoding the pro-inflammatory mediators IL-1β, TNF-α and IL-6 implicated in ischemic brain injury. HI-induced increases in mRNA levels for IκBα (a surrogate marker for NF-κB activation) were completely suppressed in animals that received AF4 prior to HI. The ability of different flavonoids found in AF4 [flavonol (quercetin) and flavan-3-ols (epicatechin)] to inhibit distinct signaling events necessary for NF-κB activation may account for the high degree of inhibition achieved by AF4 pretreatment against HI-induced NF-κB activation and the expression of genes such as IL-1β, TNF-α and IL-6 that are driven by this transcriptional regulating factor. HI also elevated mRNA levels for TLR4 that are thought to reflect the activation of pro-apoptotic signaling pathways through this receptor, resulting in neuronal cell death. These increases were suppressed in animals that received AF4. By contrast, the induction of TLR2 and EPO mRNAs levels by HI, associated with enhanced pro-survival signaling, were not suppressed by AF4. In the case of the striatum, AF4 pretreatment enhanced the induction of EPO gene expression by HI suggesting that increased production of this pro-survival cytokine may contribute to the neuroprotective effects of AF4. Similarly, the enhanced induction of mRNA levels for the potent anti-apoptotic protein XIAP by HI in AF4 pretreated animals may also play a role in the neuroprotective effects of this flavonoid enriched fraction. These transcriptional mechanisms may explain, at least in part, the need to repeatedly administer AF4 (25–50 mg/kg, once daily for at least 3 days) prior to HI in order to prevent subsequent brain damage. Lastly, pre-incubation with AF4 (1 µg/ml) protected primary cultures of mouse cortical neurons from OGD-induced death, whereas this concentration of the major AF4 constituent quercetin-3-*O*-glucoside or several major metabolites of this flavonoid were ineffective. This finding is in keeping with our hypothesis that the profound effects of oral administration of AF4 against HI-induced brain damage are achieved by co-operative, perhaps even synergistic, actions between different phenolic compounds in this fraction that interact with functionally distinct targets.

## Supporting Information

Table S1 **The Minimum Information for Publication of Quantitative Real-Time PCR Experiments (MIQE) guidelines.** This table was adapted from MIQE guidelines (Bustin SA et al (2009) Clin Chem 55: 611–622). Our compliance to the MIQE guidelines is presented in column C. (E) Essential information and (D) desirable information.
(XLS)

Table S2 **Context sequences for each primer/probe pair.** TaqMan® Gene Expression Assays were provided by Applied Biosystems Inc. (Carlsbad, California). The supplier also provided the interrogated reference sequences as well as locations of the anchor nucleotide, and the amplicon lengths. Context sequences were calculated according to established guidelines (Bustin SA et al. (2011) Clin Chem 57: 919–921). In the listed context sequences, the anchor nucleotide is highlighted in bolded text.
(XLSX)

Figure S1 **Quality analysis of extracted RNA using the Experion automated electrophoresis system.** Ten months following RNA extraction and storage, three random samples from each experimental group (Veh-Sham, Veh-HI, AF4-Sham, AF4-HI) were assessed for RNA purity using an Experion™ RNA StdSens Analysis Kit (Bio-Rad Laboratories (Canada) Ltd; Mississauga, Ontario). Samples were removed from −80°C storage and prepared according to instructions from the manufacturer. One µl of each sample was loaded for analysis. A) All samples were classified as Green (pass) or Yellow (acceptable) using established RNA quality indicator (RQI) standards (http://www.gene-quantification.org/Bio-Rad-bulletin-5761.pd). No samples were classified as Red (unacceptable). B) The virtual gel visualizing the electropherogram data shows that 28S:18S ratios are consistent between samples and that there was negligible degradation of RNA 10 months after processing and storage.
(PPTX)

Figure S2 **Determination of reference genes for qRT-PCR normalization.** Random samples of total RNA extracted from the striatum of 3 animals in each experimental group (Veh-Sham, Veh-HI, AF4-Sham, AF4-HI) were analyzed by qRT-PCR for relative quantities of mRNAs produced by three reference genes [β-Actin, Glyceraldehyde 3-Phosphate Dehydrogenase (GAPDH), and Hypoxanthine Phosphoribosyltransferase 1 (HPRT1)]. Reverse transcription, PCR amplification and fluorescence detection were performed in duplicate for each reference gene using a CFX96 Touch™ Real-Time PCR Detection System with a C1000 Touch™ thermal cycler with analysis by CFX Manager 3.0 (Bio-Rad Laboratories (Canada) Ltd; Mississauga, Ontario). A) Relative quantities of mRNA for each reference gene from each sample. B) geNorm report. Pairwise variations and M-values were calculated from the relative quantities of mRNAs for each reference gene using geNorm 3.5 software as described previously (Vandesompele J, De PK, Poppe B et al. (2002) Genome Biol 3: RESEARCH0034). β-Actin and HPRT1 both generated M-value scores of <0.5, indicating their acceptability as reference genes.
(PPTX)
